# Towards Photodynamic Image-Guided Surgery of Head and Neck Tumors: Photodynamic Priming Improves Delivery and Diagnostic Accuracy of Cetuximab-IRDye800CW

**DOI:** 10.3389/fonc.2022.853660

**Published:** 2022-06-28

**Authors:** Chanda Bhandari, John Fakhry, Menitte Eroy, Jane Junghwa Song, Kimberley Samkoe, Tayyaba Hasan, Kenneth Hoyt, Girgis Obaid

**Affiliations:** ^1^ Department of Bioengineering, University of Texas at Dallas, Richardson, TX, United States; ^2^ Thayer School of Engineering, Dartmouth College, Hanover, NH, United States; ^3^ Wellman Center for Photomedicine, Massachusetts General Hospital and Harvard Medical School, Boston, MA, United States; ^4^ Division of Health Sciences and Technology, Harvard University and Massachusetts Institute of Technology, Cambridge, MA, United States

**Keywords:** IGS, photodynamic priming, head and neck cancer, monoclonal antibodies, NIR fluorophores, tumor delivery

## Abstract

Fluorescence image-guided surgery (IGS) using antibody conjugates of the fluorophore IRDye800CW have revolutionized the surgical debulking of tumors. Cetuximab, an anti-epidermal growth factor receptor (EGFR) monoclonal antibody, conjugated to IRDye800CW (Cet-IRDye800) is the first molecular targeted antibody probe to be used for IGS in head and neck cancer patients. In addition to surgical debulking, Cetuximab-targeted photodynamic therapy (photoimmunotherapy; PIT) is emerging in the clinic as a powerful modality for head and neck tumor photodestruction. A plethora of other photoactivable agents are also in clinical trials for photodynamic-based therapies of head and neck cancer. Considering the vascular and stromal modulating effects of sub-therapeutic photodynamic therapy, namely photodynamic priming (PDP), this study explores the potential synergy between PDP and IGS for a novel photodynamic image-guided surgery (P-IGS) strategy. To the best of our knowledge, this is the first demonstration that PDP of the tumor microenvironment can augment the tumor delivery of full-length antibodies, namely Cet-IRDye800. In this study, we demonstrate a proof-of-concept that PDP primes orthotopic FaDu human head and neck tumors in mice for P-IGS by increasing the delivery of Cet-IRDye800 by up to 138.6%, by expediting its interstitial accumulation by 10.5-fold, and by increasing its fractional tumor coverage by 49.5% at 1 h following Cet-IRDye800 administration. Importantly, PDP improves the diagnostic accuracy of tumor detection by up to 264.2% with respect to vicinal salivary glands at 1 h. As such, PDP provides a time-to-surgery benefit by reducing the time to plateau 10-fold from 25.7 h to 2.5 h. We therefore propose that a pre-operative PDP regimen can expedite and augment the accuracy of IGS-mediated surgical debulking of head and neck tumors and reduce the time-to-IGS. Furthermore, this P-IGS regimen, can also enable a forward-looking post-operative protocol for the photodestruction of unresectable microscopic disease in the surgical bed. Beyond this scope, the role of PDP in the homogenous delivery of diagnostic, theranostic and therapeutic antibodies in solid tumors is of considerable significance to the wider community.

## Introduction

Head and neck cancer is the 6^th^ most common cancer worldwide and surgical resection is one of the primary interventions used in managing tumor progression (1). The current standard of care for surgical debulking of head and neck tumors relies on preoperative imaging techniques, such as computed tomography (CT), magnetic resonance (MR), and positron emission tomography (PET) imaging, in addition to intraoperative visual inspection and histological analysis. However, the correct identification of positive tumor margins using these approaches is limited, thereby frequently leading to incomplete resection (2). In a study of patients with head and neck squamous cell carcinoma having clear surgical margins (tumor ≥ 5mm from the closest surgical margin), close surgical margins (tumor 1-5 mm from the closest surgical margin) and involved surgical margins (tumor < 1mm from the closest surgical margin), 35% of patients developed tumor recurrence at the primary site (3). Tumor recurrence therefore often occurs following incomplete resection, thus requiring repeated surgeries (4). The surgical bed is oftentimes assessed histologically for residual disease, which is time consuming and inefficient (5). Furthermore, determining positive margins in the head and neck region is challenging due to the critical organs residing in close proximity to the surgical region. Moreover, with current standard of care for the identification of surgical margins, numerous satellite regions remain unnoticed, which affects the overall survival of patients (6). Therefore, there remains to be a considerable need for accurate identification of tumor boundaries and the accurate identification of surgical margins to mitigate local recurrence.

Recently, the use of fluorescently labeled ligands (*e.g.* monoclonal antibodies, antibody fragments, natural ligands etc.) that specifically bind to tumor receptors has emerged as a promising strategy to enable the precise localization of tumors and an accurate assessment of the tumor margins intraoperatively (7–9).This approach, known as fluorescence molecular image-guided surgery (IGS) was pioneered by Van Dam and colleagues using a FITC conjugate of folate, which has since been approved by the FDA in 2021 as CYTALUX™ for surgical guidance in adults with ovarian cancer (FDA Label Reference ID: 4886203) (7). Fluorescence molecular IGS has also been recently developed by Rosenthal and colleagues using IRDye800CW conjugates of antibodies, such as epidermal growth factor receptor (EGFR)-targeting Cetuximab (7, 10, 11). Cetuximab-IRDye800CW (Cet-IRDye800) has been used for fluorescence IGS in patients with head and neck cancer (ClinicalTrials.gov Identifier: NCT03134846, Identifier: NCT01987375), esophageal cancer (ClinicalTrials.gov Identifier: NCT04161560), pancreatic cancer (ClinicalTrials.gov Identifier: NCT02736578), brain malignancies (ClinicalTrials.gov Identifier: NCT02855086) and rectal cancer (ClinicalTrials.gov Identifier: NCT04638036). Multiple clinical trials led by Rosenthal and colleagues have successfully demonstrated the use of Cetuximab and Panitumumab (anti-EGFR monoclonal antibodies) conjugated to IRDye800CW for surgical resection of head and neck tumors, 95% of which overexpress EGFR (12). Consequently, they were able to precisely locate the positive margin of primary tumors as well as satellite regions, which are frequently overlooked.

We have previously demonstrated that the use of an intracellularly-activatable version of fluorescently labeled Cetuximab, along with a spectrally resolved sham IgG, can improve tumor margin delineation in orthotopic pancreatic tumors using hyperspectral IGS (13). While antibody-based IGS is a major advancement in surgical tumor debulking, the long time-intervals (typically 4 days) between probe administration and surgical debulking with IGS is a limitation (14). Strategies that shorten that duration and allow patients to undergo IGS shortly after probe administration are currently in development. One cutting edge advancement that is in early clinical development is the use of EGFR-targeting affibody molecules conjugated to IRDye800CW, such as ABY-029 (ClinicalTrials.gov Identifier: NCT02901925, NCT03154411, NCT03282461). Given its small molecular weight, shortened circulation times and faster clearance rates with respect to Cet-IRDye800, ABY-029 can be administered 1-3 h prior to fluorescence IGS.

Aside from innovations in surgical guidance in head and neck cancer, Cetuximab conjugated to another NIR dye, IRDye700DX, has also been recently approved in Japan for molecular targeted photodynamic therapy (PDT) of head and neck cancer. This approach, also known as photoimmunotherapy (PIT), is currently in Phase III trials for head and neck cancer in the US (ClinicalTrials.gov Identifier: NCT03769506). PDT is primarily an anticancer modality where photosensitizers (PSs), such as IRDye700DX are excited by a specific wavelength of light to produce cytotoxic and tumor-modulatory reactive molecular species (RMS) (15–17). Protoporphyrin IX, an intrinsic PS also used for PDT in head and neck cancer patients (ClinicalTrials.gov Identifier: NCT00978081, NCT03638622) is also in clinical trials as an intrinsic fluorescent agent for IGS of malignant gliomas (ClinicalTrials.gov Identifier: NCT02632370). This process leveraging intrinsically-generated protoporphyrin IX is oftentimes referred to as photodynamic diagnosis (PD). Photochemical internalization (PCI), a PDT-based cytosolic delivery modality has also been in clinical trials for head and neck cancer patients (NCT00993512).

Despite PDT-based approaches primarily being used for therapeutic purposes, their role extends into the realm of IGS and theranostics (18, 19). We, and others, have previously shown that therapeutic and sub-therapeutic PDT can modulate the tumor microenvironment by reducing tumor collagen density, and enhancing extravasation out of the tumor vasculature (19–23). Photodynamic priming (PDP) of the tumor microenvironment has thus led to improved tumor delivery of various nanoparticles and nanomedicines. In the clinic, PDP of pancreatic cancer has exhibited radiographic signatures that can be determined from CT images. In a study by Vincent et al, the areas having PDT-induced necrosis and PDP affected non-necrotic regions were identified through texture analysis using CT scan. The study reported statistically significant texture features pre and post PDP treatments. Also, the authors discussed that this radiomic PDP analysis helps to identify the PDP affected zones for subsequent therapies (24). To the best of our knowledge, the role of PDP in enhancing the tumor delivery of full-length antibodies, either monoclonal or polyclonal, has not been shown before for antibody targeted therapies or for antibody-based IGS.

**Figure 1:**
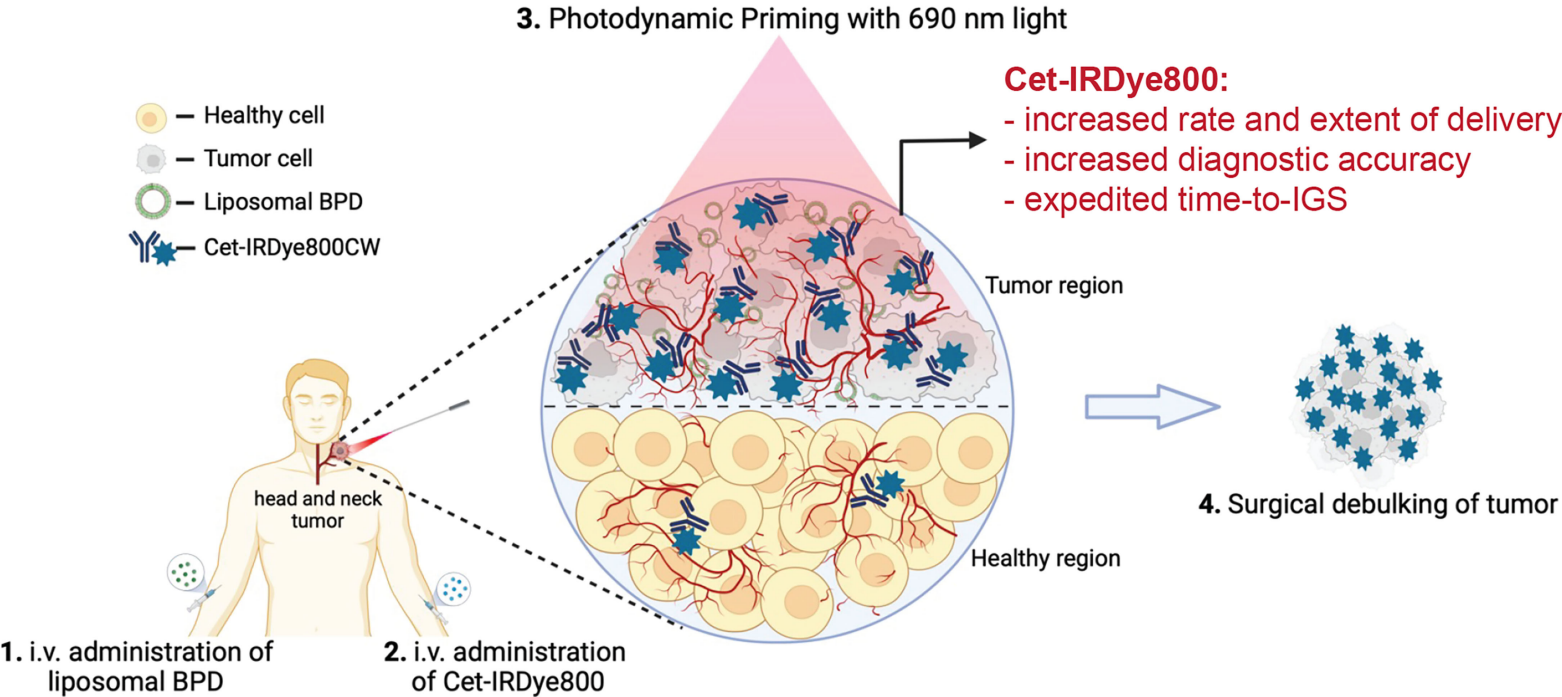
Conceptual Representation of the proposed Photodynamic Image-Guided Surgery Approach. Photodynamic Priming of the tumor microenvironment enhances the tumor delivery of Cet-IRDye800 and its accuracy in detecting tumor tissue. This approach is proposed to assist in the image-guided surgical debulking of head and neck tumors, amongst others. This Figure was created from Biorender.com using an Academic License.

Considering the emerging role of PDT-based approaches in head and neck tumors, as well as the role of Cet-IRDye800 in IGS, an opportune synergy between PDP and IGS is presented here. In this study, we modulate the microenvironment of orthotopic FaDu human head and neck tumors in mice with PDP using PEG-modified liposomal benzoporphyrin derivative (BPD) that mimics the approved photonanomedicine Visudyne (lactose, egg phosphatidylglycerol, dimyristoyl phosphatidylcholine, ascorbyl palmitate and butylated hydroxytoluene; FDA Label Reference ID: 3927523). PDP is performed immediately after intravenous administration of the in-house mimetic of the clinical conjugate Cet-IRDye800. The impact of PDP on the dynamic tumor delivery and accuracy of Cet-IRDye800 in orthotopic FaDu human head and neck tumors is explored and presented as a rational and opportune combination of two emergent technologies for the further improved management of head and neck cancers using this presented Photodynamic Image-Guided Surgery (P-IGS) approach (**Figure 1**).

PDT itself holds significant potential in treating primary tumors prior to surgery, as has been shown for pancreatic cancer whereby neoadjuvant PDT enabled surgical resection of an originally unresectable patient tumor (25). However, it must be noted that in Phase IIa clinical trials for antibody-targeted PDT (PIT), an overall response rate of 43% was observed (95 percent CI 25.5-62.6) (ClinicalTrials.gov Identifier: NCT02422979). Thus, PDT, although highly promising, cannot be used as a monotherapy. While this P-IGS approach is proposed as an enabling tool to assist IGS, it does also enable the pursuit of adjuvant PDT of the surgical bed following resection to eliminate microscopic, unresectable disease. This post-surgical PDT approach has been already attempted in gliomas and was found to be safe, but results of an efficacy study are still pending (26).

## Materials and Methods

### Synthesis and Characterization of Cetuximab-IRDye800CW (Cet-IRdye800)

Cetuximab (Erbitux, Ely Lily; 2 mgml^-1^, 145781.6 g/mol as per FASTA sequence analysis) was conjugated to IRDye800CW (IRDye800CW-NHS Ester; Li-COR, 1166.20 g/mol) by amide coupling. A stock solution of IRDye800CW-NHS Ester (10 mg·ml^-1^) was prepared in anhydrous Dimethyl Sulfoxide (DMSO; Sigma-Aldrich) and added to Cetuximab (2 mg·ml^-1^ in phosphate buffer) at a quantity corresponding to a 10-fold molar excess of IRDye800CW to each molecule of Cetuximab. The mixture was thoroughly mixed using orbital rotation for 24h at room temperature. After conjugation, the Cet-IRDye800 was purified by removing unconjugated dye using PD-10 Desalting Columns (Cytivia) pre-equilibrated with sterile 1X DPBS (Corning). Purified Cet-IRDye800 was collected and concentrated in a 30kDa ultrafiltration tube (EMD Millipore) by centrifugation at 2,500 xg for 20 min at 4°C. The conjugated Cet-IRDye800CW was then stored at 4°C in the dark.

### Synthesis of Liposomal Benzoporphyrin Derivative

PEG-coated liposomal benzoporphyrin derivative (Lipo-BPD) was used for PDP in this study. Briefly, lipo-BPD was synthesized as per the conventional thin film hydration method. All lipids were purchased from Avanti Polar Lipids. Firstly, DPPC, cholesterol, DSPE-mPEG_2000_ and Benzoporphyrin Derivative Monoacid Ring A (US Pharmacopeia) were mixed together in chloroform at a molar ratio of 0.66:0.289:0.03:0.01 (27). 1X DPBS was used to hydrate the thin film at 42°C and extrusion was performed through 100 nm polycarbonate membranes (Whatman).

### Characterization of Cet-IRDye800 and Liposomal BPD

The concentration of Cetuximab and IRDye800CW in the conjugate was measured using UV-Visible spectrophotometry (Thermo Evolution 350 Spectrophotometer) using molar extinction coefficients of ε_280nm_= 217,315 M^-1^cm^-1^ and ε_774nm_ = 240,000 M^-1^cm^-1^ in PBS, respectively, with a correction factor of 0.03 for IRDye800CW at 280 nm. Similarly, the BPD concentration in the liposomes was measured using UV-Visible spectrophotometry with a molar extinction coefficient of ε_687nm_ = 34,895 M^-1^cm^-1^ in DMSO. The hydrodynamic diameter and polydispersity index of liposomal BPD was measured in triplicates with 2μl of liposomal BPD dispersed in 1ml of 1X DPBS (Corning) using a Zetasizer Pro (Malvern) dynamic light scattering instrument.

### Orthotopic FaDu Tumor Xenograft Implantation

All procedures were performed in accordance with Institutional Animal Care and Use Committee (IACUC) protocols. Male athymic Swiss Nude Mice (20 g, 6 wk old) purchased from Charles River, were maintained in a fully equipped animal facility in compliance with the NIH guide for the Care and Use of Laboratory animals at the University of Texas at Dallas Vivarium. FaDu cells were purchased from ATCC (HTB-43) as mycoplasma-free and authenticated cells, and were used according to ATCC protocols. Cells were cultured in DMEM (Corning) containing 10% Fetal Bovine Serum (R&D Systems). 7.5 x 10^5^ FaDu cells suspended in 20 μl sterile 1X DPBS were implanted orthotopically transcervically into the floor of the mouth according to previously published procedures (28, 29). 15 d following tumor implantation, the tumors reached a diameter of *ca.* 5-8 mm, as determined by ultrasound imaging (VisualSonics Vevo 3100) and were subsequently used for Cet-IRDye800 imaging and PDP.

### Longitudinal Imaging and P-IGS

**Figure 2:**
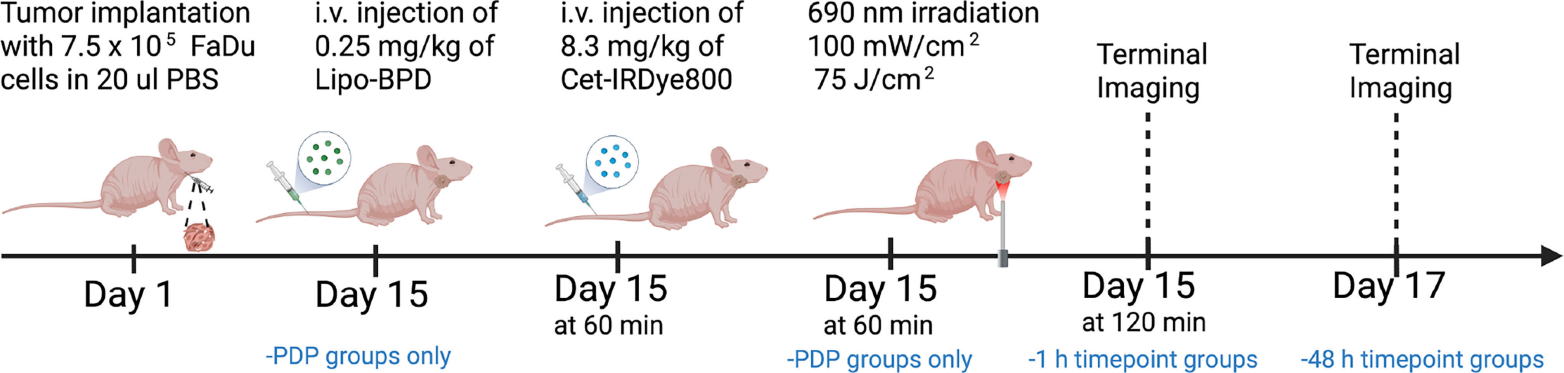
Schematic representation of the procedures and timeline of the *in vivo* studies involving the PDP process and imaging of Cet-IRDye800 in FaDu human head and neck tumors.

Mice bearing FaDu tumors were divided into four arms as per the time of euthanasia following intravenous Cet-IRDye800 administration and whether or not they were subject to PDP: 1) 1 h no PDP group (n=10), 2) 48h no PDP group (n=6), 3) 1 h PDP group (n=10), and 4) 48 h PDP group (n=6). **Figure 2** is a schematic representation of the procedures and respective timeline of the *in vivo* studies. All groups were intravenously injected with 8.3 mg.kg^-1^ of Cet-IRDye800 which corresponds to the human equivalent dose of 25 mg·m^-2^ (ClinicalTrials.gov Identifiers: NCT01987375, Identifiers: NCT02415881). Mice were imaged prior to and post (1, 3, 6, 9, 12, 24 and 48 h) intravenous administration. For the PDP groups, sub-therapeutic PDT was performed using a previously published dose immediately following Cet-IRDye800 administration (19). Briefly, liposomal BPD was intravenously injected at 0.25 mg·kg^-1^ BPD equivalent 30 min before Cet-IRDye800 administration and 1h before 690 nm photoactivation. 30 min following Cet-IRDye800 administration, the tumor region was exposed to the sub-therapeutic dose of 75 J·cm^-2^ of 690 nm laser light (Modulight) at an irradiance of 100 mW·cm^-2^ (19). Mice were given subcutaneous Meloxicam SR analgesia (ZooPharm) following PDP to minimize discomfort. All longitudinal and terminal imaging of Cet-IRDye800 was performed using the LI-COR PEARL Imaging system. Terminal imaging was performed after euthanasia and removal of the skin covering the tumor region, followed by imaging of the surgical bed after the tumor was harvested. The tumor was then bisected and cryopreserved in OCT (Tissue-Plus™ O.C.T. Compound; Fisher Scientific) for histological analysis along with the salivary glands, tongue tissue and hind leg muscle. The frozen tissue was cryosectioned at 20 μm sections using the Cryostat-Leica CM1860, stained with hematoxylin and eosin (H&E), and imaged using the Olympus VS120 Virtual Slide Microscope. The LI-COR Odyssey DLx Slide Scanner was also used to visualize Cet-IRDye800 in all harvested tissues.

### Image Analysis

Longitudinal and terminal mean Cet-IRDye800 tumor signals were quantified using the LI-COR PEARL Imaging System software and were corrected using distant skin regions. Tissue cryosections imaged with the Odyssey DLx Slide Scanner used to quantify fractional tumor coverage and mean tumor signals of Cet-IRDye800 in the cryosections using Otsu thresholding on ImageJ. Receiver Operating Characteristic (ROC) curves were generated using 100 point mean Cet-IRDye800 measurements from all tumor, salivary gland, tongue and muscle tissue. Point measurements were performed using ImageJ and ROC curves were generated using GraphPad Prism v9.2.0. Contrast-based diagnostic accuracy analyses (contrast-variance) were performed according to the following equation (30):


$$contrast-to-variance=\frac{meantumorsignals-meannormaltissuesignals}{\sqrt{\sum \left(tumorstandarddeviation+normalstandarddeviation\right)}}$$

All statistical analyses including *t-*tests and one-way analysis of variance (ANOVA) tests were performed using GraphPad Prism v9.2.0.

## Results

### Synthesis and Characterization of Cet-IRDye800 and Liposomal BPD

Cetuximab and IRDye800CW were conjugated using amide coupling and spectrophotometry revealed that 3 IRDye800CW molecules were bound to each molecule of Cetuximab. The mean hydrodynamic diameter of liposomal BPD was found to be 137.6 ± 0.6 nm with a polydispersity index of 0.09 ± 0.01, which resembles the physical properties of the liposomal BPD construct previously shown to facilitate PDP *in vivo* (135 nm with a polydispersity index of 0.04) (19).

### PDP Improves the Delivery and Diagnostic Accuracy of Cet-IRDye800 and Expedites Time-to-IGS

**Figure 3:**
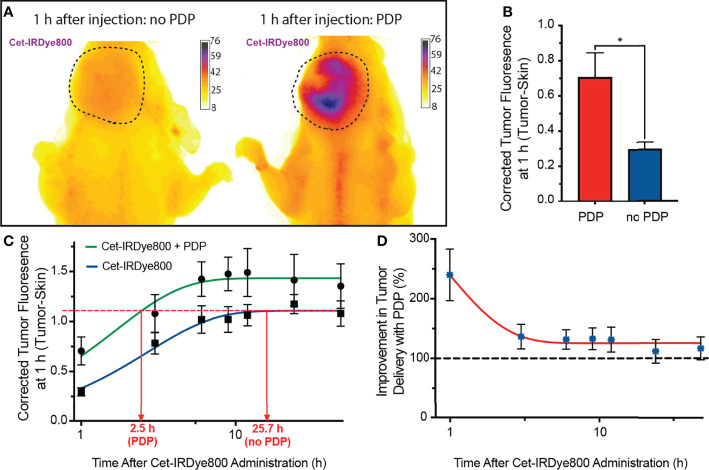
**(A)** Representative fluorescence images of Cet-IRDye800 accumulation in orthotopic FaDu head and neck tumors with (right) and without (left) PDP at 1 h following probe administration. **(B)** Quantitation of tumor accumulation of Cet-IRDye800 at 1 h following probe administration. **(C)** Longitudinal fluorescence imaging of Cet-IRDye800 within tumors with and without PDP. **(D)** Longitudinal non-invasive quantitation of Cet-IRDye800 tumor content in PDP-treated tumors with respect to tumors that were not subject to PDP. (Values are mean ±S.E.M.; statistical analysis was performed using a two-tailed *t-*test (b); *P≤ 0.05).

The delivery of Cet-IRDye800 to the tumors, either with or without PDP, was measured using longitudinal *in vivo* imaging in an orthotopic murine model of FaDu human head and neck cancer. Histological tissue analysis shows no evidence of necrosis in irradiated tumors, nearby salivary glands, nearby tongue tissue and distant muscle, thereby verifying that the PDP dose used is in fact below the therapeutic threshold (**Figure S1**). **Figure 3A** shows representative images of tumors in mice 1 h post administration of Cet-IRDye800 where visibly greater signals were observed in the tumor that was subjected to PDP. FaDu tumor accumulation of Cet-IRdye800 was increased by up to 138.6% ± 47.3% with PDP at 1 h following intravenous administration (**Figure 3B**). Without PDP, the tumor accumulation of Cet-IRdye800 reached a plateau at 25.7 h following intravenous administration (**Figure 3C**). However, with PDP, the same degree of tumor accumulation is achieved in only 2.5 h, corresponding to a 10.5-fold shorter duration, as revealed by a nonlinear regression analysis (**Figure 3C**). Should this same degree of shortened duration till surgery be translated to humans, patients would be ready for IGS using Cet-IRDye800 on the same day as administration. Interestingly, it was found that the improvement in Cet-IRDye800 tumor delivery was highest at 1 h following administration (**Figure 3D**). By 48 h after administration, Cet-IRDye800 tumor accumulation was found to be only 25.5% ± 20.6% greater than the accumulation in tumors without PDP. The rate of Cet-IRDye800 accumulation in tumors were also found to be 2.5-fold greater with PDP (k = 1.04/h) than without PDP (k = 0.42/h). Given the rapid enhancement of the delivery of Cet-IRDye800 into the tumor by PDP at 1h, it is likely that the microenvironmental modulation occurs immediately after PDP. Considering that the tumor uptake of Cet-IRDye800 without PDP is only 25.5% less than with PDP at 48 h, PDP appears to have a greater influence on the kinetics of tumor uptake of Cet-IRDye800. This observation warrants future critical studies that evaluate the effects at varying PDP dose products (administered PS x light fluence) as well as PS-light intervals on the pharmacokinetics of Cet-IRDye800. In addition, the accumulation of Cet-IRDye800 in healthy skin and muscle was quantified at 1h. Although PDP appeared to increase Cet-IRDye800 in the skin by 15.7% (**Figure S3a**), the increase in skin uptake with PDP was not statistically significant (P=0.2866). It is conceivable, however, that scattered light during the process of topical tumor illumination may augment healthy skin accumulation of Cet-IRDye800. This further emphasizes the need for molecular specificity of the PDP probes to selectively modulate tumor vasculature and tumor stroma.

**Figure 4:**
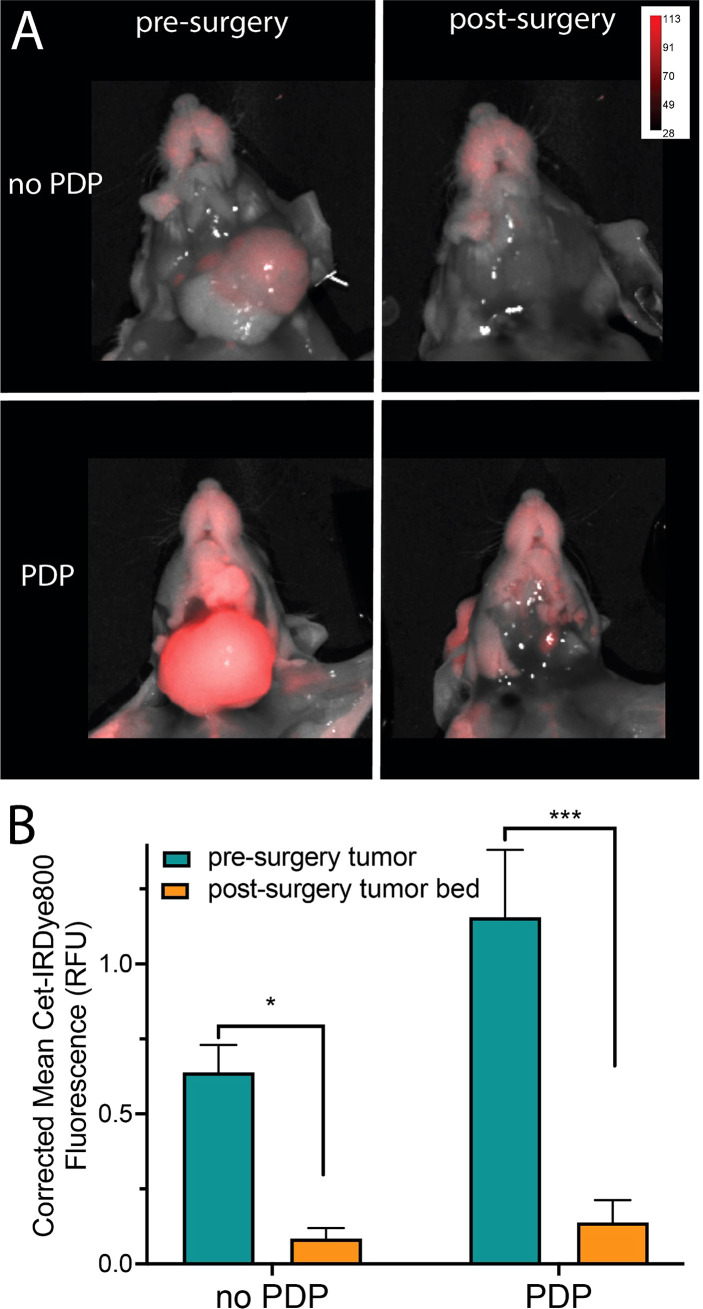
**(A)** Representative fluorescence and white light combined images of tumors subjected to PDP (bottom) and no PDP (top) pre- (left) and post-surgical debulking (right) of the tumor. **(B)** Comparison of mean Cet-IRDye800 fluorescence intensity in the tumors with and without PDP, both before and after surgical debulking of the tumors. (Values are mean ± S.E.M.; statistical analysis was performed using one-way ANOVA with a Tukey post-test **(B)**; *P ≤ 0.05, ***P ≤ 0.005).

**Figure 5:**
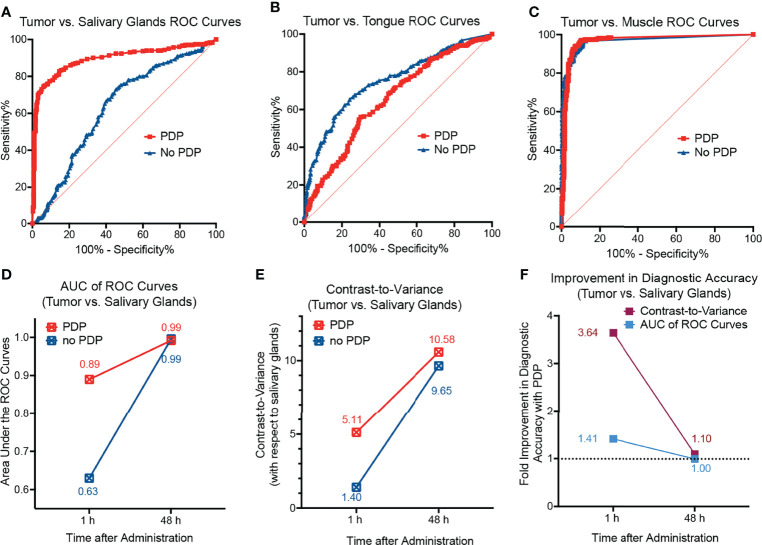
Receiver operating characteristic (ROC) curves of tumors with respect to **(A)** salivary glands, **(B)** the tongue, and **(C)** healthy muscle tissue from the hind leg of mice 1 h after administration of Cet-IRDye800 with and without PDP. **(D)** The diagnostic accuracy with respect to salivary glands as depicted by the AUC analyses of the ROC curves suggests that PDP increases the diagnostic accuracy at 1 h after administration. **(E)** The diagnostic accuracy with respect to salivary glands as depicted by the measure of contrast-to-variance suggests that PDP increases the diagnostic accuracy at both 1 h and 48 h after administration. **(F)** The percentage improvement of the diagnostic accuracy with respect to salivary glands with PDP at both 1 h and 48 h after administration.

After 1 h following PDP, the mice were euthanized, the skin was removed, and fluorescence imaging of the tumor region was performed (**Figure 4A** **).** Although limited in that there is no real-time image guidance, a pseudo-simulation of IGS was performed by imaging of the surgical bed before and after surgical debulking of the primary tumor, both with and without PDP. Tumor signals in the surgical bed were significantly reduced following surgical debulking both with and without PDP; however, the degree of signal reduction was 1.8-fold greater following surgical debulking of PDP-treated tumors than in tumors without PDP (**Figure 4B**). Given the limited nature of *in vivo* preclinical imaging systems, the true value of PDP for IGS can only be actualized when using real-time fluorescence imaging systems with optical magnifications that allow for precision resection of tumor tissue. The data presented in **Figure 2** demonstrates the degree in the reduction of Cet-IRDye800 probe signals between PDP treated and non-PDP treated tumors after surgical debulking. The closest that pre-clinical studies on fluorescence-based image-guided surgery have reached with respect to simulating image-guided surgery in mice is using white light resection followed by ex vivo fluorescence analysis of the accuracy of probe accumulation (31, 32).. Even if microscopy-enable real-time intraoperative imaging capabilities were widely available for preclinical image-guided surgery in mouse models, their resolution and spatial accuracy are not representative of the clinical systems used for fluorescence image guided surgery in patients. Furthermore, the anatomy of mouse tumor models does not faithfully represent that of patient tumors, nor does their pathobiology (*eg.* infiltrative potential). The Cet-IRDye800 signals in the surgical bed following tumor debulking do not originate from unresected disease, as these baseline signals are in fact lower than the non-specific Cet-IRDye800 signals in healthy muscle tissue (**Figure S3B**). As such, true real-time (photodynamic) image-guided surgery would need a sophisticated microscopic setup in addition to a more infiltrative tumor model than the one presented here. Given these caveats, the power of the PDP approach we present here lies in its ability to increase the accuracy of tumor detection by Cet-IRDye800 with respect to healthy tissue. As such, the widely accepted gold standard used for assessing the potential for improving the accuracy of image-guided surgery in a preclinical and clinical setting is using fluorescence-based imaging of the tissue sections *ex vivo* followed by receiver operating characteristic curve (ROC) quantitation of specificity and sensitivity (10, 30, 31, 33–36). This is demonstrated in **Figure 5**, where ROC curves represent the sensitivity and specificity of tumor tissue detection by Cet-IRDye800 with and without PDP. Considering the importance of anatomical relevance in the comparison, PDP provides a clear advantage in increasing the accuracy of Cet-IRDye800 detection of tumor tissue with respect to the salivary glands at 1 h following probe administration (**Figure 5A** **)**. Area under the ROC curve analyses summarized in **Table 1** show that the accuracy of Cet-IRDye800 tumor detection in PDP treated tumors is 41.3% greater than in untreated tumors 1 h following probe administration (**Figures 5D, F** ). With respect to another measure of diagnostic accuracy, the contrast-to-variance ratio, PDP improved the diagnostic accuracy of Cet-IRDye800 with respect to salivary glands by 264.2% (3.6-fold) at 1 h and by 9.6% (1.096-fold) at 48 h after administration (**Figures 5E, F** **)**. There does, however, appear to be a 15% compromise in Cet-IRDye800 tumor detection with respect to the tongue (**Figure 5B**; **Table 1**). It worth noting that this experiment was performed on head and neck tumors where the tumor was grown on the floor of mouth in close proximity to the tongue. With an untargeted PDT construct using the unfocused laser beam in this proof-of-concept study, there is a greater possibility of 690 nm light penetrating through the tumor into the tongue region, thereby promoting PDP-mediated Cet-IRDye800 delivery into the healthy tongue tissue as well. Although this is resolved by 48 h after probe administration (**Figure S2**), it does emphasize the importance of spatial precision in tumor tissue irradiation or molecular precision in PDP of tumor tissue and/or tumor vasculature when using antibody conjugates or derivatives thereof. To increase the diagnostic of the tumor with respect to the tongue, one of the potential options can be using pre-PDP imaging (*e.g.* computed tomography) to allow for PDP treatment planning and guidance for 690 nm irradiation. Another example is the use of anti-VEGFR-2 antibodies to target tumor vasculature specifically to ensure a greater degree of precision in priming the tumor for IGS. However, these remain to be the focus of future studies to identify the optimal combination of light irradiation parameters and PS formulation. PDP provides no additional benefit for the diagnostic accuracy of Cet-IRDye800 tumor detection with respect to distant muscle from the hind leg at 1h **(** **Figure 5C** and **Table 1** **).** This is likely due to the fact that the diagnostic accuracy without PDP with respect to distant muscle (AUC of ROC curve = 0.97) is already the highest of all tissue assessed, and thus it will be the least likely to increase further with PDP.

**Table 1:** Summary of the diagnostic accuracy of Cet-IRDye800 with and without the photodynamic priming (PDP) approach.

	AUC for no PDP(mean ± S.E.M.)	AUC for PDP(mean ± S.E.M.)
**Tumor vs. Salivary Glands**	0.63 ± 0.02***	0.89 ± 0.01***
**Tumor vs. Tongue**	0.75 ± 0.02***	0.65 ± 0.02***
**Tumor vs. Muscle**	0.97 ± 0.01***	0.97 ± 0.01***

***P < 0.0001; statistical significance represents significance of each individual rea under the receiver operating characteristic curve with respect to tumor versus healthy tissue. Statistical significance was calculated using the Wilson/Brown test using GraphPad Prism v9.2.0.

Area under curve (AUC) analyses of the receiver operating characteristic (ROC) curves for the tumors subjected to PDP and no-PDP conditions at 1 h following administration of Cet-IRDye800 with respect to the salivary glands, tongue tissue and hind leg muscle tissue.

**Figure 6:**
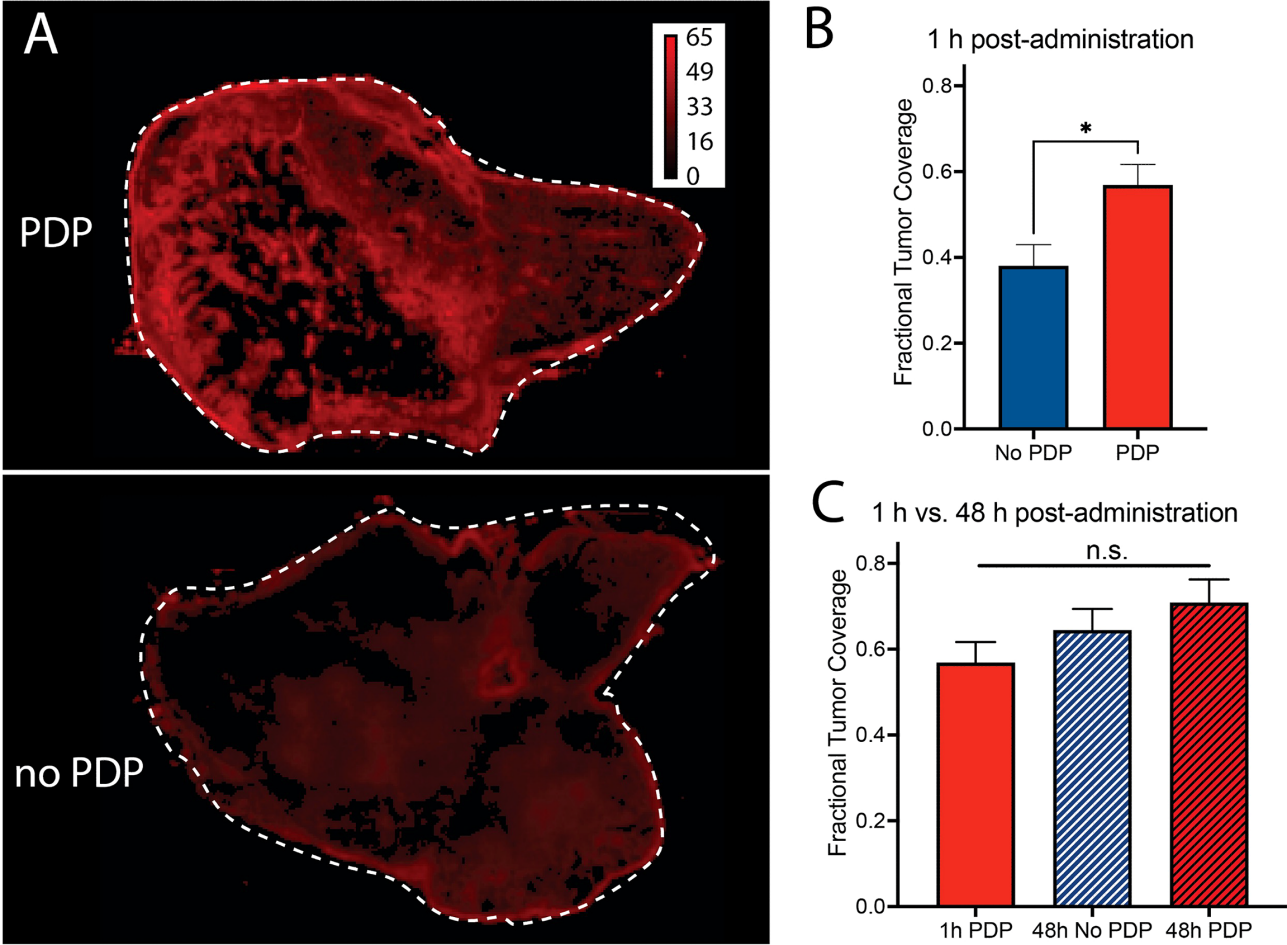
**(A, B)** Fractional tumor coverage of Cet-IRDye800 in bisected tumor cross-sections is significantly increased with PDP at 1 h following intravenous administration. At 48 h, **(C)** no significant improvements in Cet-IRDye800 fractional tumor coverage are observed with respect to 1 h PDP. (Values are mean ± S.E.M.; statistical analysis was performed using a two-tailed *t* test **(B)** and One-Way ANOVA with a Tukey Post-Test **(C)**; *P ≤ 0.05).

Finally, the fractional tumor coverage of Cet-IRDye800 in bisected tumor cross-sections with PDP and without PDP was quantified, which is representative of the homogeneity in tumor tissue distribution of the probe (**Figure 6A**). At 1 h following Cet-IRDye800 administration, the fractional tumor coverage was enhanced by 49.5% with PDP, whereby PDP improved the fractional tumor coverage from 0.38 to 0.57 (**Figure 6B**). The increased fractional tumor coverage at 1 h following Cet-IRDye800 administration provided by PDP was not significantly improved by waiting for an additional 47 h, either with or without PDP treatment **(** **Figure 6C** **).** This suggests that the homogeneity of tumor tissue coverage by Cet-IRDye800 at 1h post-administration is optimal when the tumors were primed by PDP, thereby enabling IGS at shorter time-periods with greater probability of complete tumor resection.

## Discussion

Both Cetuximab-based fluorescence IGS and Cetuximab-targeted PDT are emerging as powerful tools in the clinical management of head and neck cancers. While both valuable in their own right, IGS of primary tumors is most likely to provide the greatest benefit to patients when followed up with intraoperative PDT of the surgical bed to eliminate residual disease. Seeing the potential synergy between PDT with IGS, we hereby propose Photodynamic IGS (P-IGS), whereby a pre-operative sub-therapeutic PDT protocol (PDP), augments and expedites the delivery of Cet-IRDye800, and also increases its accuracy of tumor tissue detection. To the best of our knowledge, this is the first demonstration that PDP of solid tumors enhances the rate and extent of delivery of full-length antibodies. Although the effects of enhanced delivery and other systemic effects have been noted in the field of PDT for some time, only recently has the concept of PDP begun to be codified (19, 37, 38). A better understanding of PDP is evolving and is likely to lead to a planned enhancement of not only drug and agent delivery but also local and systemic effects of a mechanistic synergy with secondary theranostic interventions. With future developments aimed at increasing the spatial precision of P-IGS with both image-guided photoactivation procedures and molecular targeted PSs, it is conceivable that the benefit of synergizing PDP with IGS can be maximized and further extended to post-surgical eradication of residual microscopic disease in the surgical bed.

### Future Perspectives and a Pathway to Photodynamic Image-Guided Surgery

It is well established that sub-therapeutic PDT, PDP, can significantly modulate the tumor microenvironment to facilitate the delivery of nanomedicines (19–23). This modulation has been associated with temporary increases in tumor vasculature permeability (19, 22, 23), a decrease in cancer-associated fibroblast activity (39) and a decrease in collagen density within a solid tumor (20, 21). In this study, we also now demonstrate that the modulation of the tumor microenvironment using PDP can promote and expedite the delivery of Cet-IRDye800 in an orthotopic head and neck model. In this study, the PDP effect is confined to the tumor microenvironment by controlling the area of irradiation. As such, the diagnostic accuracy with respect to nearby salivary gland tissue is significantly increased at 1 h after administration. This P-IGS protocol therefore holds significant potential in reducing the time-to-surgery by a 10-fold.

In general, it is well established that the efficacy of PDT (and PDP) depends on the extent of PS localization within the tumor during irradiation. The clinical PS BPD that’s used in this study is hydrophobic and, as such, liposomal BPD (including the clinical liposome Visudyne) is more widely used as a result of its greater accumulation within the tumor. The tumor selectivity of such liposomal formulations of hydrophobic PSs depending solely on passive preferential tumor delivery through the Enhanced Permeability and Retention (EPR) effect (16, 40). However, tumor tissue selectivity of liposomal PS formulations may not be sufficient in providing the greatest precision for PDT or PDP. Thus control over the area of irradiation becomes the primary determinant of spatial accuracy when using such passively targeted liposomal PS formulations, as we have shown in this study. With the clinical advances of photoimmunotherapy (PIT; antibody targeted PDT), molecular precision in photodamage is emerging as a clinical reality, even though it had been in pre-clinical development for decades (16, 40). By adopting strategies that target the tumor vasculature, tumor cells and cancer-associated fibroblasts, molecular precision in PDP hold significant potential in further improving the diagnostic accuracy of molecular probes, such as Cet-IRDye800, for P-IGS. This can be particularly powerful when coupled with a tumor-activatable approach to further augment the specificity of tumor detection and tumor damage (13, 41–43).

**Figure 7:**
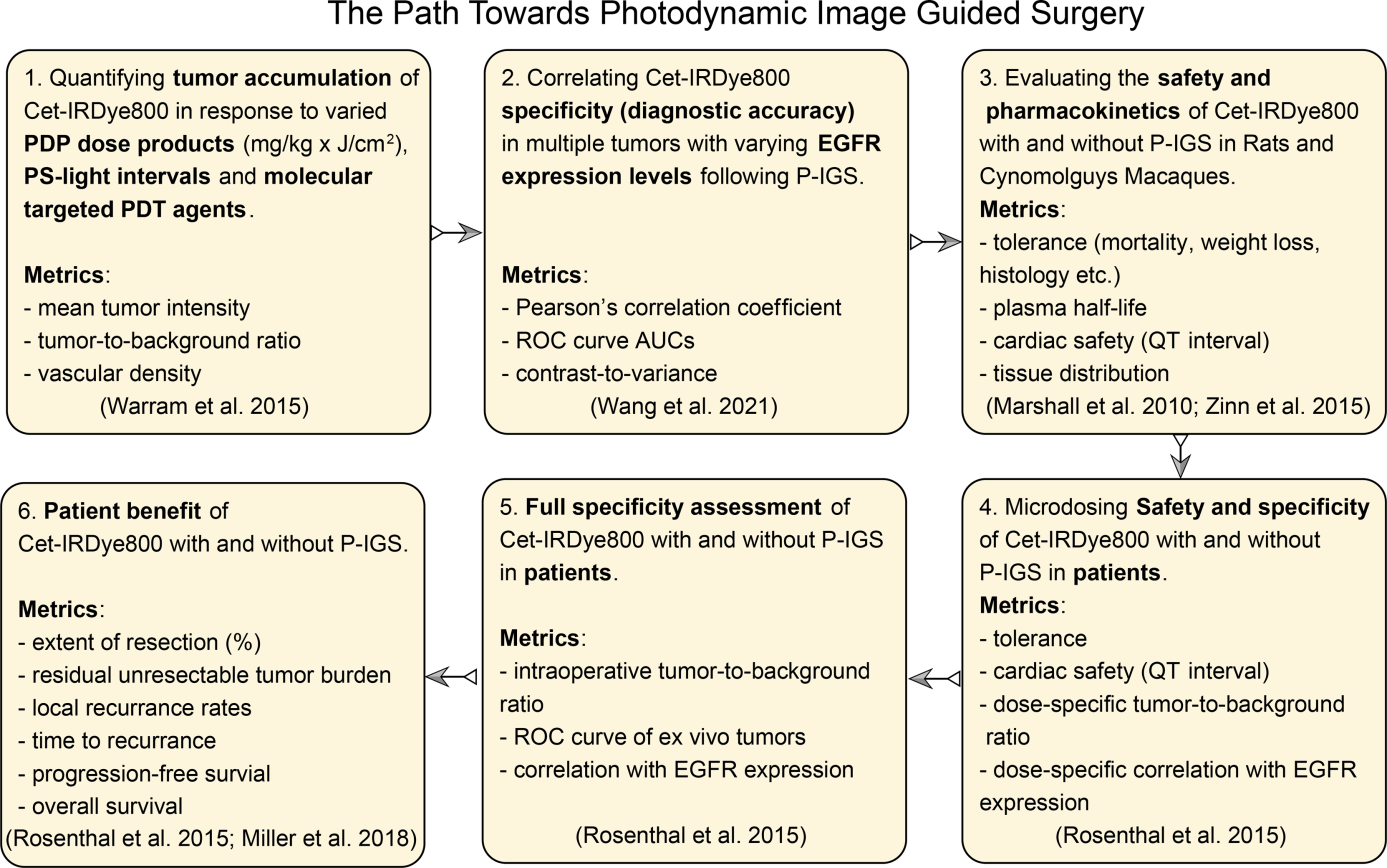
The path towards photodynamic image-guided surgery proposed, including anticipated necessary metrics, in accordance with the published path of clinical translation of Cet-IRDye800 and related IGS probes (10, 30, 44–46).

The evidence presented in this proof-of-concept warrants the pursuit of P-IGS. The pathway to P-IGS will comprise multiple stages of pre-clinical and clinical development, which can be modeled on the paths taken for the clinical translation of molecular probes for IGS (10, 11, 30, 44–46). **Figure 7** is a multi-stage pathway proposed for the further development of the P-IGS approach, which is modeled on these aforementioned paths used for translating Cet-IRDye800 and related probes. The first objective of the future studies would be to identify the molecular targeted PDP agent, PDP dosimetry and PS-light interval which provides the greatest diagnostic accuracy to Cet-IRDye800 and the fastest time-to-IGS. The next stage would be to assess this PDP + Cet-IRDye800 regimen in tumors with varying EGFR expression levels and to correlate the diagnostic accuracy with EGFR expression. A particular emphasis on tumors with extensive vascular and stromal heterogeneity would be important in order to assess the capacity for PDP to improve the homogeneity of Cet-IRDye800 distribution and tumor margin delineation. Following these assessments, the safety and pharmacokinetics of Cet-IRDye800 with and without the optimized PDP regimen will be tested in animal (*eg.* rats and Cynomolguys Macaques) and human subjects. The dose-dependent specificity of Cet-IRDye800 with and without the optimized PDP regimen would also need full assessment in human subjects, followed by the assessment of how this P-IGS approach may provide maximal patient tumor resection, reduce local recurrence rates, reduce the time to recurrence and prolong progression-free and overall survival. This P-IGS approach holds considerable potential for maximizing the extent and accuracy of resection within clinically-relevant time frames, while enabling future approaches for post-surgical PDT of the surgical bed, thereby offering head and neck cancer patient with previously unattainable survival benefit.

## Data Availability Statement

The original contributions presented in the study are included in the article/**Supplementary Material**. Further inquiries can be directed to the corresponding author.

## Ethics Statement

The animal study was reviewed and approved by UT Dallas IACUC Committee.

## Author Contributions

CB: Developing Experimental Methodologies and Primary Experiments, Writing-Original Draft, Writing-Review and Editing. JF: Primary Experiments, Data analysis, Writing-Original Draft. ME: Data analysis, Formal Analysis. JS: Primary Experiments. KS: Supervision, Data analysis, Writing: Review and Editing. TH: Writing: Review and Editing. KH: Primary Experiments, Supervision, Writing: Review and Editing, Funding Acquisition and GO: Conceptualization, Supervision, Investigation, Developing Experimental Methodologies, Formal Analysis, Writing: Original Draft, Writing: Review and Editing, Resources, Project Administration, Funding Acquisition. All authors have contributed to this study and accepted responsibility for the entire submission.

## Funding

This work was supported by the National Institutes of Health [R00CA215301 to GO and R01EB025841 to KH] and the Cancer Prevention and Research Institute of Texas Award [RP180670 to KH].

## Conflict of Interest

The authors declare that the research was conducted in the absence of any commercial or financial relationships that could be construed as a potential conflict of interest.

## Publisher’s Note

All claims expressed in this article are solely those of the authors and do not necessarily represent those of their affiliated organizations, or those of the publisher, the editors and the reviewers. Any product that may be evaluated in this article, or claim that may be made by its manufacturer, is not guaranteed or endorsed by the publisher.
