# Supplementary material for: Towards Photodynamic Image-Guided Surgery of Head and Neck Tumors: Photodynamic Priming Improves Delivery and Diagnostic Accuracy of Cetuximab-IRDye800CW
Source: Front Oncol. 2022 Jun 28;12:853660. doi: 10.3389/fonc.2022.853660 (PMC9273965; doi:10.3389/fonc.2022.853660)
Supplement: Supplementary file 1 [file DataSheet_1.docx]

Supplementary Material

## Supplementary Figures

##
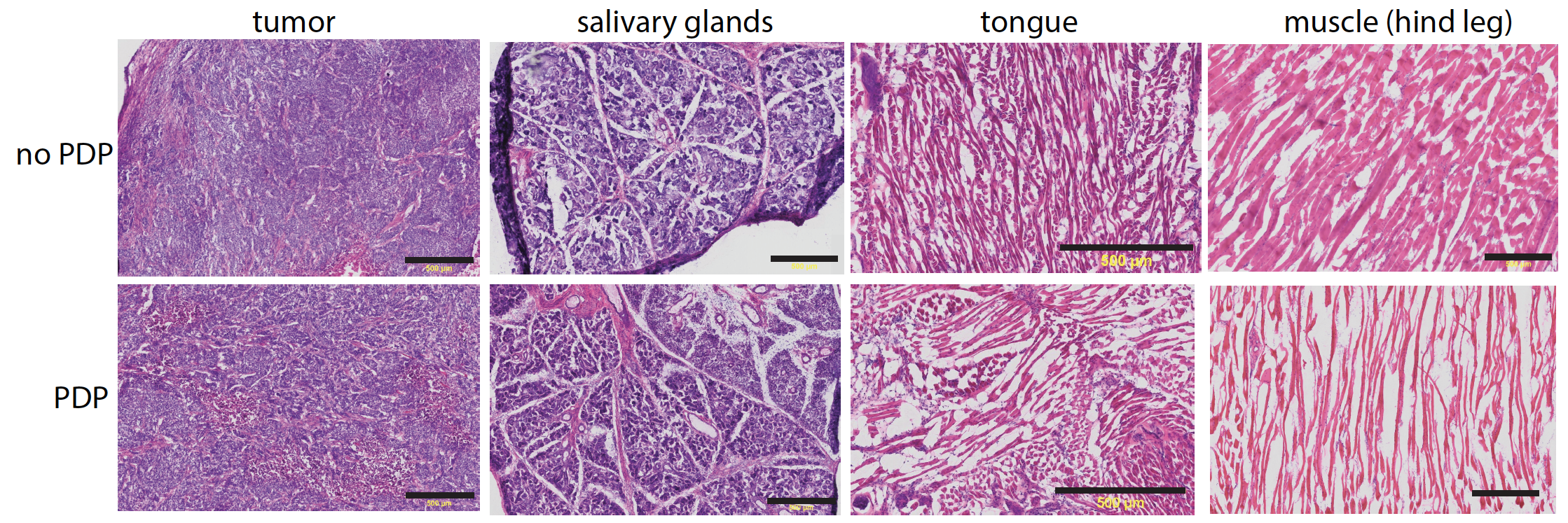
 Supplementary Figure 1. Representative H&E images of the tumor, salivary glands, tongue and muscle from the hind leg 48 h after administration of Cet-IRDye800 with and without PDP. No evidence of tissue damage is observed with PDP. (Scale bars are 500 $\boldsymbol{\mu}$m)

##
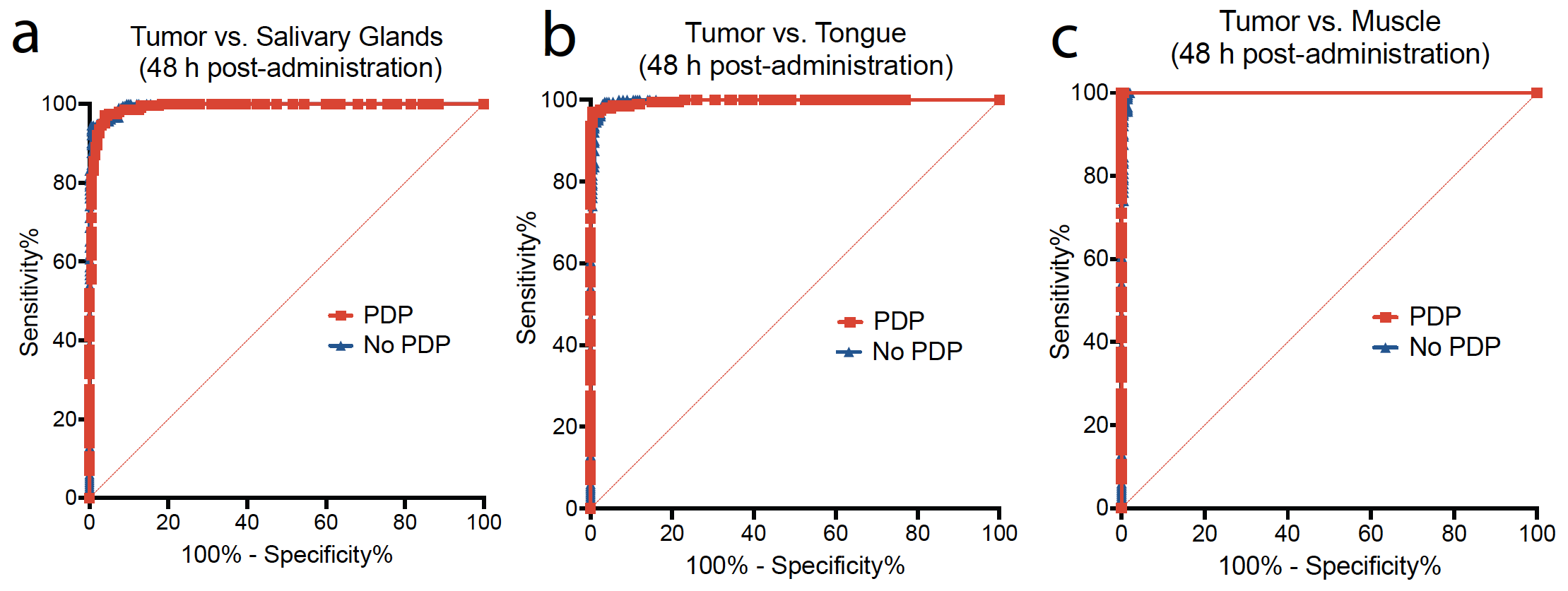
Supplementary Figure 2. Receiver Operating Characteristic (ROC) curves of tumors with respect to a) salivary glands, b) the tongue, and c) healthy muscle tissue from the hind leg of mice 48 h after administration of Cet-IRDye800 with and without PDP.

Table S1: Summary of the diagnostic accuracy of Cet-IRDye800 with and without the photodynamic priming (PDP) approach. Area under curve (AUC) analyses of the receiver operating characteristic (ROC) curves for the tumors subjected to PDP and no-PDP conditions at 48 h following administration of Cet-IRDye800 with respect to the salivary glands, tongue tissue and hind leg muscle tissue.

|  | **Diagnostic Accuracy:**  **AUC for no-PDP**  **(mean ± S.E.M.)** | **Diagnostic Accuracy:**  **AUC for PDP**  **(mean ± S.E.M.)** |
| --- | --- | --- |
| **Tumor vs. Salivary Glands** | 0.996 ± 0.002*** | 0.992 ± 0.003*** |
| **Tumor vs. Tongue** | 0.997 ± 0.000*** | 0.997 ± 0.002*** |
| **Tumor vs. Muscle** | 0.998 ± 1.000*** | 1.000 ± 0.000*** |

***** = P<0.0001; statistical significance represents significance of each individual rea under the receiver operating characteristic curve with respect to tumor versus healthy tissue. Statistical significance was calculated using the Wilson/Brown test using GraphPad Prism v9.2.0.**

**Supplementary Figure 3: a) Accumulation of Cet-IRDye800 with and without PDP in normal skin and muscle tissue. b) Cet-IRDye800 signals in the surgical bed post-resection and in nearby muscle tissue (Values are mean ± S.E.M.; statistical analysis was performed using one-way ANOVA with a Tukey post-test.)**
